# Lactylation in ischemic brain injury—Metabolic mechanisms, neuroinflammation, and therapeutic targets: A review

**DOI:** 10.17305/bb.2025.12955

**Published:** 2025-10-01

**Authors:** Xinchen Ji, Jing Lu, Ke Wang, Yan Guo, Dexi Zhao, Miao Liu

**Affiliations:** 1 Taizhou Hospital of Traditional Chinese Medicine, Jiangsu, China; 2 Research Center of Traditional Chinese Medicine, The Affiliated Hospital to Changchun University of Chinese Medicine, Changchun, China; 3 Department of Rehabilitation, The Affiliated Hospital to Changchun University of Chinese Medicine, Changchun, China; 4 School of Panax Notoginseng Medicine, Wenshan University, Wenshan, China; 5 Department of Encephalopathy, The Affiliated Hospital to Changchun University of Chinese Medicine, Changchun, China; 6 Nantong Hospital of Traditional Chinese Medicine, Jiangsu, China

**Keywords:** Lactylation modification, cerebral ischemic injury, epigenetic modification, hypoxia-reperfusion, treatment strategies

## Abstract

Cerebral ischemic injury, a major cause of mortality and disability, results from reduced or interrupted blood flow to the brain, most commonly in ischemic stroke. Insufficient oxygen and nutrient supply disrupts cellular metabolism, leading to neuronal death, neurological dysfunction, and lasting impairments. Current therapeutic strategies, including thrombolysis, mechanical thrombectomy, and anticoagulation, primarily aim to restore perfusion and provide neuroprotection by preserving the ischemic penumbra. While these interventions can partially rescue viable tissue in the acute phase, their effectiveness is constrained by narrow therapeutic windows, low recanalization rates, and contraindications, leaving significant unmet clinical needs. Consequently, the search for novel, targeted approaches has become a central focus of ischemic stroke research. Recent discoveries have identified lactylation, a newly recognized post-translational modification derived from lactate, as a key regulator of gene expression, protein function, and metabolic reprogramming. Once regarded as a simple glycolytic byproduct, lactate is now known to act as both an alternative energy substrate and a signaling molecule, influencing neuronal metabolism, antioxidant defense, and inflammatory responses. In ischemic brain injury, lactylation modifications of histone and non-histone proteins may either protect neurons—by supporting energy homeostasis, regulating stress-responsive genes, and suppressing apoptosis—or exacerbate injury through neuroinflammation, excitotoxicity, and immune evasion. Evidence indicates that the outcomes of lactylation depend on lactate concentration, timing of accumulation, cell type, and the balance between “writer” and “eraser” enzymes. Therefore, lactylation emerges as a promising yet complex therapeutic target in cerebral ischemia. Modulating lactate metabolism and its downstream modifications offers new opportunities to expand the therapeutic window, attenuate neuronal injury, and improve recovery. This review summarizes the molecular mechanisms linking lactate and lactylation to ischemic injury, highlights current contradictions in experimental findings, and explores the potential of targeting lactylation pathways for innovative treatment strategies.

## Introduction

**Figure 1.**
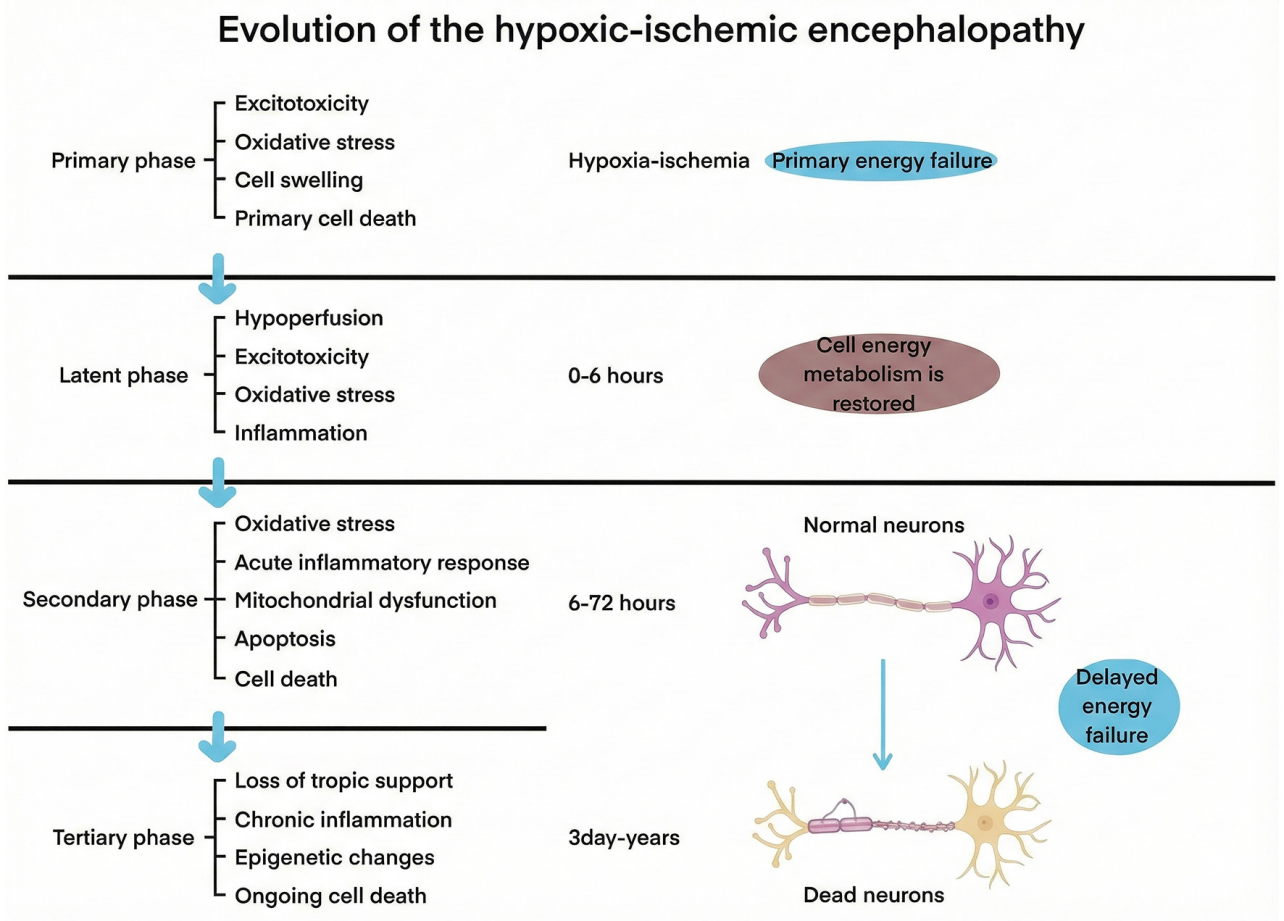
**The progression of ischemic encephalopathy.** Ischemic brain injury develops in four distinct stages. The first stage is marked by primary energy failure during hypoxic-ischemic events, which triggers detrimental effects, including ATP-dependent pump blockade, lactic acidosis, calcium ion buildup, excitatory amino acid release, toxic edema formation, and necrosis in the brain’s most vulnerable regions. This is followed by a latent phase (pre-Stage 2), known as the energy recovery phase during resuscitation. In the subsequent 6–72 h (Stage 2), the energy consumption process of the brain reoccurs in areas with greater resistance, maintaining excitotoxicity. This stage is characterized by a substantial influx of calcium ions, increased activation of neuronal NOS, oxidative stress, and mitochondrial dysfunction, ultimately leading to secondary energy failure and neuronal death through caspase pathway activation. Mitochondrial deterioration and an acute inflammatory response are key features of this stage, resulting in apoptosis. The third stage is defined by persistent inflammation and epigenetic changes. During this phase, oxidative stress causes direct damage to the central nervous system and activates a cascade of inflammatory responses, thereby accelerating the progression of Stage 3. Prolonged inflammation exacerbates the damage.

In recent years, many countries have reported an increase in the incidence, mortality, and disability rates associated with stroke, with ischemic stroke constituting 62.4% of all stroke cases [1–3]. This condition occurs when cerebral blood vessels become narrowed or obstructed, leading to a reduced blood supply to the brain and subsequent tissue necrosis. Chronic ischemia can result in permanent neuronal damage and neurological impairments (Figure 1), significantly affecting patients’ quality of life and imposing a substantial burden on families and society [4–6]. Currently, pharmacological and surgical reperfusion therapies are the primary effective treatments. The core strategy of these neuroprotective therapies is to preserve the ischemic penumbra surrounding the necrotic core, where blood flow is relatively less compromised. Early reperfusion therapy aims to salvage brain tissue within this ischemic penumbra. However, prolonged ischemia can cause the necrotic core to expand, leading to irreversible neuronal damage in surrounding tissue. The limited therapeutic window, coupled with low recanalization rates and numerous contraindications to thrombolysis, diminishes the clinical efficacy of reperfusion therapy [7–9].

Recent studies underscore the significant role of cellular metabolic reprogramming, particularly glycolysis, in the progression of ischemic and hypoxic diseases following ischemia and hypoxia, a phenomenon that has garnered considerable attention [10, 11]. Lactic acid, once regarded merely as a byproduct of glycolysis, is now recognized as a vital metabolite. It serves not only as an energy source for various tissues, including skeletal muscle, heart, brain, and cancer cells but also functions as a signaling molecule involved in immune regulation, fat mobilization, wound healing, and the maintenance of cellular homeostasis [12–14]. Additionally, recent research indicates that lactic acid plays a role in the repair process after ischemic brain injury, with exogenous lactic acid supplementation aiding in the reduction of ischemic brain damage [11, 15–17].

Lactylation, a post-translational modification of proteins, has emerged as a critical mechanism in the regulation of cellular metabolism. This modification affects gene expression, protein activity, and processes related to the development of ischemic diseases [18–20]. Lactylation can occur on both histones and non-histones within cells, with enzymes such as histone acetyltransferases (e.g., p300, CBP) and deacetylases (e.g., HDACs) playing essential roles in regulating these modifications. Targeting these enzymes may offer novel therapeutic strategies for the treatment of ischemic diseases [21–23].

This review aims to explore the molecular mechanisms underlying lactylation modification, its role in the progression of ischemic brain injury, and potential therapeutic strategies that target lactylation, given its critical importance in ischemic disease biology. As research on lactylation in cerebral ischemic injury (in both neonatal and adult models) is still in its infancy, this review will also discuss future prospects for leveraging lactylation and its regulatory pathways as innovative therapeutic approaches.

Recent studies underscore the pivotal role of cellular metabolic reprogramming, particularly glycolysis, in the advancement of ischemic and hypoxic diseases. This phenomenon has garnered substantial attention [10, 11]. Lactic acid, once regarded merely as a byproduct of glycolysis, is now recognized as a vital metabolite. It not only acts as an energy source for various tissues—including skeletal muscle, heart, brain, and cancer cells—but also functions as a signaling molecule involved in immune regulation, fat mobilization, wound repair, and the maintenance of cellular homeostasis [12–14]. Moreover, recent research indicates that lactic acid plays a significant role in the repair process following ischemic brain injury, with exogenous supplementation of lactic acid demonstrating efficacy in reducing ischemic brain damage [11, 15–17].

Lactylation, a post-translational modification of proteins, has emerged as a crucial mechanism in the regulation of cellular metabolism. This modification affects gene expression, protein activity, and processes associated with the development of ischemic diseases [18–20]. Lactylation can occur on both histones and non-histones within cells, with enzymes such as histone acetyltransferases (e.g., p300, CBP) and deacetylases (e.g., HDACs) playing essential roles in modulating these modifications. Targeting these enzymes may offer innovative therapeutic strategies for the treatment of ischemic diseases [21–23].

This review aims to elucidate the molecular mechanisms of lactylation modification, its role in the progression of ischemic brain injury, and potential therapeutic strategies that target lactylation, given its critical significance in ischemic disease biology. As research on lactylation in cerebral ischemic injury (in both neonatal and adult models) remains in its nascent stages, this review will also explore future prospects for leveraging lactylation and its regulatory pathways as novel therapeutic approaches.

## Methods

### Literature search strategy

To conduct a thorough review of the literature on lactylation modification and ischemic brain injury, a systematic search strategy was employed. We searched the following databases: PubMed, Google Scholar, and Web of Science, covering the period from January 1, 2000 to June 30, 2025. The search terms included “lactylation AND stroke,” “lactylation AND ischemic brain injury,” and “lactate AND cerebral ischemia.” The literature search was restricted to articles published in English, focusing exclusively on peer-reviewed journal articles.

Inclusion criteria consisted of: (1) studies investigating the relationship between lactylation modification and ischemic brain injury; (2) both clinical and experimental studies that provided complete experimental data; (3) high-quality review articles that offered comprehensive background information and references; and (4) peer-reviewed journal articles. Exclusion criteria included: (1) studies that did not directly explore the role of lactylation or lactate in ischemic brain injury; (2) conference papers and studies lacking complete data; and (3) studies characterized by poor quality or inadequate experimental design.

### Lactic acid and lactylation modifications

Lactic acid, an essential metabolic byproduct, is primarily generated in the cytoplasm and plays a crucial role in glycolysis. Under conditions of hypoxia or heightened metabolic demand, glucose is converted into pyruvate via glycolysis. In scenarios of limited oxygen availability, particularly during ischemic injuries, pyruvate cannot enter the mitochondria for oxidative phosphorylation and is instead converted to lactate. This conversion is catalyzed by lactate dehydrogenase (LDH), which facilitates the reduction of pyruvate to lactate while oxidizing NADH to NAD+, thereby ensuring the continuation of glycolysis [24–26].

**Figure 2.**
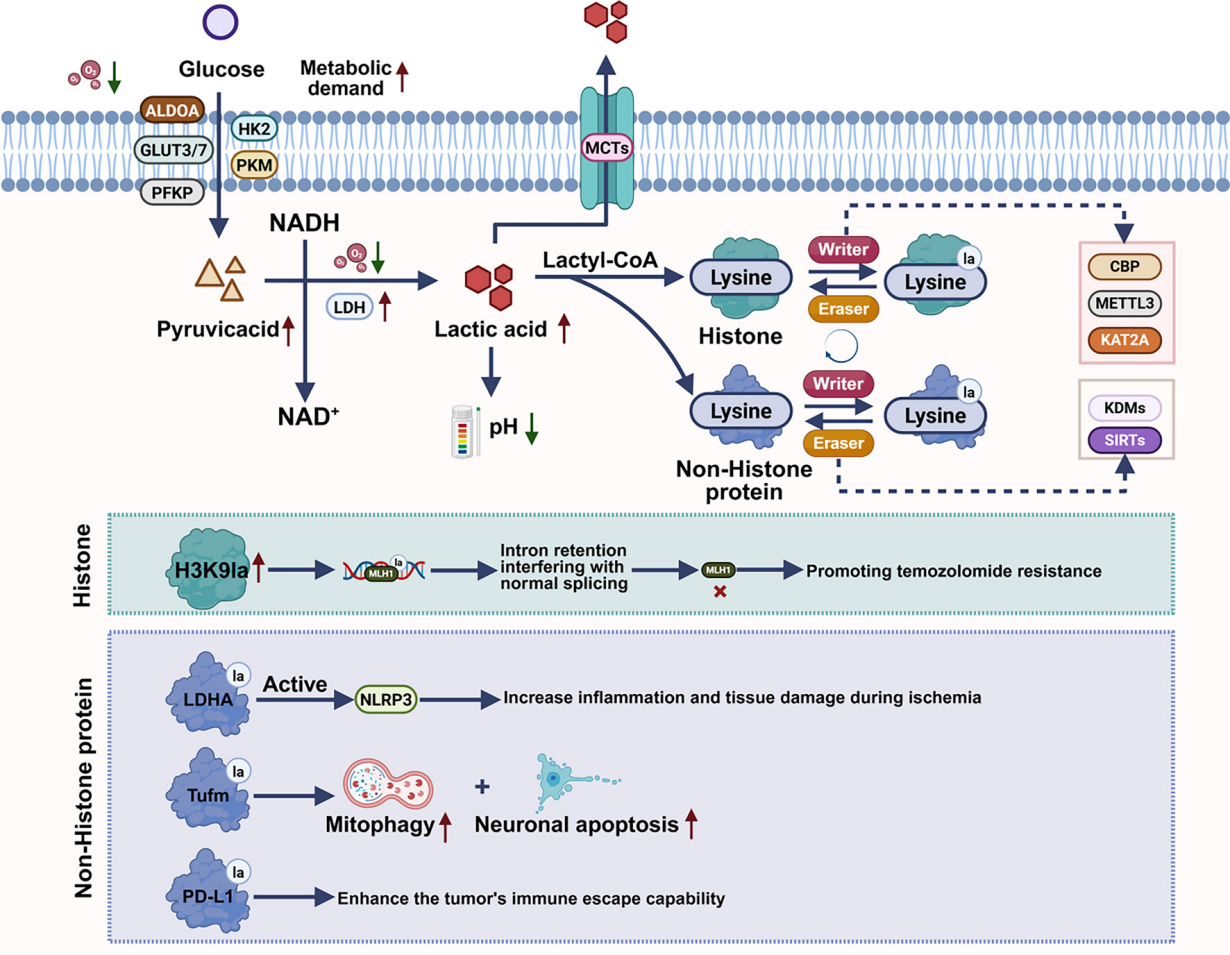
**Diagram depicting the processes of glycolysis, lactate production, and lactylation modifications.** Glycolysis generates lactate from pyruvate via LDH, with excess lactate exported by MCTs to balance pH. Lactate can form lactyl-CoA, driving lysine lactylation (Kla) on histone and non-histone proteins. Kla is regulated by specific “writers” (CBP, METTL3, KAT2A) and “erasers” (HDACs, KDMs, SIRTs). Histone lactylation (e.g., H3K9la) alters splicing and promotes temozolomide resistance, while non-histone lactylation enhances inflammation (NLRP3), mitophagy and neuronal apoptosis (Tufm), and immune evasion (PD-L1). This highlights lactate–lactylation as a key regulator in cancer and ischemic injury. Abbreviations: ALDOA: Aldolase A; GLUT1/GLUT3: Glucose transporter 1/3; HK2: Hexokinase 2; PKM: Pyruvate kinase M; PFKP: Phosphofructokinase, platelet type; LDH: Lactate dehydrogenase; NAD^+^/NADH: Nicotinamide adenine dinucleotide (oxidized/reduced); MCTs: Monocarboxylate transporters; Lactyl-CoA: Lactyl-coenzyme A; H3K9la: Histone H3 lysine 9 lactylation; MLH1: MutL homolog 1; LDHA: Lactate dehydrogenase A; NLRP3: NOD-like receptor family pyrin domain-containing 3; Tufm: Tu translation elongation factor, mitochondrial; PD-L1: Programmed death-ligand 1; CBP: CREB-binding protein; METTL3: Methyltransferase-like 3; KAT2A: Lysine acetyltransferase 2A (GCN5); KDMs: Lysine demethylases; SIRTs: Sirtuins.

The production of lactate is closely linked to the energy requirements of the cell and is regulated by various essential proteins. Key regulators of lactate production include LDH, which directly influences lactate generation [27, 28], as well as several enzymes that facilitate glycolysis, including glucose transporter proteins (GLUT), Pyruvate kinase M1/2 (PKM), Hexokinase 2 (HK2), Aldolase A (ALDOA), and Phosphofructokinase platelet type (PFKP), among others [29–32]. For instance, the expression and activity of LDH are often elevated in tumor cells, accelerating lactate production [33–35]. GLUT family members, such as GLUT1 and GLUT3, enhance glucose uptake, ensuring an adequate supply of substrates necessary for glycolysis [36, 37]. Accumulation of lactate leads to a decrease in local pH, prompting cells to utilize specific transporters, such as monocarboxylate transporters (MCTs), to export lactate, thereby preventing excessive accumulation and helping to maintain intracellular pH balance [38–41]. The coordinated actions of these proteins sustain the equilibrium of lactate production, regulating both the cell’s metabolic state and its capacity to adapt to environmental changes (Figure 2).

Lactylation, a significant post-translational modification, regulates protein function and structure through the attachment of a lactyl group to the side chain of an amino acid. The first identification of lysine lactylation (Kla) occurred in 2019, marking a post-translational modification derived from lactate. This modification is evolutionarily conserved and prevalent across various cell types [42–44]. Within the cell, lactylation is initiated when lactate—produced endogenously or introduced exogenously—reaches a critical concentration threshold [45–47].

The lactylation process commences with lactate serving as the substrate, which is subsequently converted into lactyl-CoA [48, 49]. Lactyl-CoA then acts as a substrate for a specific group of acetyltransferases that transfer the lactyl group to lysine residues in both histones and non-histones, leading to alterations in protein structure and function. These enzymes, often referred to as “writers,” include P300/CREB-binding protein (CBP) [50–52], Methyltransferase-like 3 (METTL3) [53–55], and Lysine acetyltransferase 2A (KAT2A), among others [56–59]. Conversely, “erasers,” such as histone deacetylases (HDACs) [60–63], Lysine demethylases (KDMs) [64], and Sirtuins (SIRTs) [65–67], remove the lactyl group from lysine residues on proteins, thereby preventing prolonged effects of lactylation. The collaborative actions of both “writers” and “erasers” regulate the equilibrium of protein lactylation, facilitating stable functional modifications within the body (Figure 2).

Proteins that undergo lactylation modifications include both histones and non-histones (Figure 2) [68–73]. Initially discovered in histones, lactylation modifications have since driven increased research into the regulation of histones, particularly in neurological diseases. For example, histone H3K9 lactylation (H3K9la) has been found to promote temozolomide resistance in glioblastoma by causing MutL homolog-1 (MLH1) intron retention via LUC7-like 2 (LUC7L2). Specifically, temozolomide treatment results in an upregulation of H3K9la levels in glioblastoma cells. This modification occurs within the intronic region of the MLH1 gene, leading to intron retention and interference with normal splicing, thereby impairing MLH1 function and facilitating temozolomide resistance [74]. Furthermore, pharmacological inhibition of glycolysis can reduce H3K9la, consequently increasing the sensitivity of glioblastoma cells to temozolomide.

Although initial lactylation research primarily focused on histones, more recent studies have expanded to examine non-histone proteins [75]. Lactate and lactylation play vital protective roles in ischemic stroke by maintaining neuronal function and promoting cell survival. Lactate serves as an alternative energy source for neurons, sustaining cellular energy and preventing neuronal death during ischemia. Lactylation modifications, particularly in histones, regulate gene expression that may aid cells in adapting to ischemic stress. Recent studies suggest that lactylation in microglia modulates neuroinflammation, contributing to neuroprotection during ischemic events [76]. Additionally, lactylation in ischemic stroke has been shown to regulate genes involved in neuronal survival, enhancing resilience to injury. However, excessive lactate accumulation and dysregulated lactylation can exacerbate neuronal injury. High lactate levels lead to acidosis, excitotoxicity, and mitochondrial dysfunction. Dysregulated lactylation further promotes inflammation and neuronal death. Fang et al. demonstrated that LDHA-induced lactylation activates the NLRP3 inflammasome, increasing inflammation and tissue damage in ischemia [77]. Weng et al. [78] showed that lactylation of the Tufm protein induces mitophagy and neuronal apoptosis, contributing to neuronal injury. Additionally, Yang et al. [76] highlighted how lactylation of Programmed death-ligand 1 (PD-L1) can enhance immune evasion in tumors, illustrating lactylation’s dual, context-dependent effects.

### The role of lactylation in ischemic brain injury has been a topic of interest

Initial studies have indicated an increase in lactate levels following ischemic brain injury, with these elevated levels potentially serving as biomarkers [79]. Subsequent research has demonstrated that while lactate levels rise after ischemic brain injury, the supplementation of exogenous lactate can effectively mitigate damage, suggesting a protective role for lactate in such injuries [80–82]. Despite this, the precise mechanism through which lactate exerts its protective effects remained unclear for an extended period. Recent findings have illuminated how lactate influences the neuronal GPR81 protein, regulates brain angiogenesis during development, and promotes recovery from hypoxic-ischemic injury [11]. Furthermore, lactate has been shown to protect both neurons and astrocytes from ischemic damage by regulating calcium levels [83]. While these studies suggest that lactate treatment enhances protective factors and diminishes harmful ones, the mechanisms by which lactate, as a metabolic byproduct, regulates these factors had not been previously explored. It was only after the introduction of the concept of lactylation that researchers began to understand the intricate relationship between lactate and ischemic brain injury.

Yao et al. [84] were the first to demonstrate that lactylation modifications are enhanced in ischemic brain injury. They investigated Kla in cortical proteins from the cerebral ischemia–reperfusion injury (CIRI) model in adult rats, noting an increase in lactylation modifications associated with ischemic brain injury. A total of 1003 lactylation sites were identified across 469 proteins. However, their study did not provide mechanistic insights nor establish whether lactylation modifications have protective or detrimental effects on ischemic brain injury.

Sun et al. [85] provided a comprehensive examination of the mechanisms by which lactylation modifications affect ischemic brain injury. They identified that MeCP2, a key transcriptional regulator, undergoes lactylation, which serves as a protective mechanism against neuronal death induced by stroke. Mechanistically, lactylation at the K210/K249 sites of MeCP2 suppresses the expression of apoptosis-related genes, such as Programmed Cell Death Protein 4 (Pdcd4) and Phospholipase A2 Group VI (Pla2g6), thereby reducing neuronal apoptosis. Additionally, HDAC3 and p300 were found to be crucial enzymes regulating the lactylation of MeCP2 following a stroke. Their findings demonstrated that lactate alleviates ischemic brain injury by modulating protein lactylation modifications within neuronal cells, elucidating specific sites and regulatory pathways involved.

Numerous studies challenge the previously held view that lactate functions solely as a protective factor [86–88]. Mitochondrial transfer, which refers to the movement of mitochondria between cells, plays a critical role in protecting against ischemic brain injury by providing energy and enhancing the function of damaged neurons. Zhou et al. demonstrated that lactylation modification regulates mitochondrial transfer, subsequently influencing the outcome of ischemic brain injury. Their research identified low-density lipoprotein receptor-related protein-1 (LRP1) as a key surface receptor involved in endocytosis and signal transduction, which regulates essential cellular processes such as survival, differentiation, and proliferation. In a mouse model of ischemic stroke, inhibiting LRP1 in astrocytes reduced mitochondrial transfer to injured neurons and worsened ischemia–reperfusion injury. Mechanistically, LRP1 in astrocytes facilitates mitochondrial transfer to neurons by decreasing lactate production and ADP-ribosylation factor 1 (ARF1) lactylation. Furthermore, LRP1 suppresses glucose uptake, glycolysis, and lactate production, ultimately resulting in a reduction of ARF1 lactylation [89]. While this study indicated that lactate inhibits mitochondrial transfer and exacerbates neuronal damage, it did not involve direct lactate application to animals or cells with ischemic brain injury. Given the multiple mechanisms at play, mitochondrial transfer represents only one protective factor, making it impossible to draw definitive conclusions regarding whether lactate or lactylation modifications are beneficial or harmful in ischemic brain injury. The only conclusion that can be drawn is that increased lactylation modification in astrocytes may worsen ischemic brain injury.

Research has also shown that lymphocyte cytosolic protein 1 (LCP1) is upregulated in ischemic brain injury models. Silencing LCP1 significantly reduced neurological deficits, infarct size, and brain water content in middle cerebral artery occlusion (MCAO) models in adult rats, while also decreasing cell apoptosis. Both total lactylation and LCP1 lactylation levels were markedly elevated in cerebral infarction, both *in vivo* and *in vitro*. Treatment with 2-Deoxy-D-glucose (2-DG) resulted in a significant reduction in LCP1 lactylation. In conclusion, inhibiting glycolysis lowered LCP1 lactylation and facilitated LCP1 degradation, ultimately reducing the progression of cerebral infarction [90]. Xiong et al. [91] conducted a study demonstrating the effectiveness of inhibiting glycolysis in reducing ischemic brain injury. Their findings were validated through an *in vivo* model, which contradicted the conclusions drawn by Sun et al. [85]. Specifically, their research showed that inhibiting LDHA or glycolysis, which reduces lactate production, resulted in significant reductions in brain damage in ischemic stroke mice. However, additional lactate supplementation exacerbated brain injury, possibly due to its association with neuronal death and the activation of A1 astrocytes. Increased lactate levels during ischemia may facilitate the formation of protein Kla, whereas post-reperfusion lactate treatment does not influence the Kla levels of neuroprotective brain proteins. Moreover, pharmacologically inhibiting lactate production or blocking its transport into neurons led to a notable reduction in Kla protein levels in ischemic brains. Further analysis of MCAO results in astrocyte-specific LDHA knockout mice showed that cKO mice exhibited lower Kla protein levels compared to the control group, alongside a decrease in brain infarction volume. Inhibiting the formation of protein Kla using the antagonist A-485, which targets the writer p300, significantly reduced neuronal death and neuroglial activation in brain ischemia. This intervention also lowered Kla protein levels, prolonging the reperfusion window and improving functional recovery in ischemic stroke. These findings suggest that lactate produced by astrocytes exacerbates ischemic brain injury by promoting the formation of protein Kla. The study underscores two essential points: 1) lactate produced by astrocytes plays a critical role in ischemic brain injury and 2) inhibiting glycolysis can help alleviate ischemic brain injury. This conclusion directly contradicts the findings of Sun et al. [85]. A closer examination of both studies reveals differences in drug administration timing: one study applied the drug 24 h before surgery, while the other administered it 40 min post-surgery. These discrepancies in experimental conditions cannot fully account for the conflicting conclusions. Additionally, while Sun et al. [85] found that exogenous lactate supplementation alleviated ischemic brain injury, Pan et al. observed that lactate supplementation worsened ischemic brain injury under the same experimental conditions [92]. The numerous contradictory conclusions cannot be solely attributed to experimental conditions, suggesting that further research is necessary to clarify the underlying reasons for these discrepancies.

The study conducted by Pan et al. is particularly notable, as it highlights the significant reduction of lactate and Kla protein levels in ischemic brain tissue of mice following electroacupuncture (EA) pretreatment, a therapeutic approach combining traditional Chinese medicine techniques. This reduction was associated with decreased astrocyte activation and less neuronal damage and death. However, the study’s interpretation of traditional medicine mechanisms through a Western medical framework introduces unnecessary complexity. The authors did not elaborate on how EA regulates the reduction in lactylation levels, nor did they clarify how this reduction contributes to the alleviation of ischemic brain injury. In contrast, certain traditional Chinese herbal formulas have been shown to modulate lactylation modifications and offer protective effects against ischemic brain injury. For instance, Song et al. found that Buyang Huanwu Decoction (BHD) alleviates ischemic brain injury [93]. While earlier studies primarily focused on neuronal cells, their research shifted to endothelial cells, demonstrating that BHD inhibits glycolysis and apoptosis by suppressing pan-Kla and H3K18la protein levels, as well as Apoptotic protease activating factor 1 (Apaf-1) transcriptional activity. This action helps prevent the progression of ischemic brain injury. A notable limitation of their study is the uncertainty regarding whether the effects of BHD are solely reliant on lactylation modification regulation in endothelial cells, as it remains unclear whether BHD can directly affect neuronal cells.

Ischemic brain injury and tumor cells both rely on glycolysis for energy production, consequently generating significant amounts of lactate, which represents a shared characteristic in their energy metabolism. However, notable discrepancies exist between ischemic brain injury and tumor cells. For instance, while glycolysis promotes tumor progression in cancer cells, ischemic brain injury has resulted in varied and sometimes contradictory conclusions. This contrast is also evident in lactylation modifications, where the effects are inconsistent not only across different cell types but even within the same cell type. Furthermore, despite utilizing identical models and drug interventions, diverse outcomes have been observed, indicating that further, more comprehensive research is necessary to fully understand the role of lactylation modifications in ischemic brain injury.

### Lactylation as a regulator of neuroinflammation in brain injury

Recent studies have established that histone lactylation is pivotal in modulating neuroinflammatory responses, particularly in microglial cells. Histone H3 lysine 9 lactylation (H3K9la) promotes M1-type pro-inflammatory polarization of microglia through the activation of the TNF-α signaling pathway. This effect can be mitigated by inhibiting the histone acetyltransferase P300 or the lactate-producing enzyme LDHA [94]. In Alzheimer’s disease, a H4K12la–PKM2 positive feedback loop exacerbates microglial activation and neurodegeneration, underscoring the role of lactylation in amplifying immune responses [95]. Similarly, in Parkinson’s disease models, H3K9la enhances SLC7A11 expression, which contributes to glutamate toxicity and microglial dysfunction [96]. These findings indicate that lactylation extends beyond energy metabolism, implicating it in chronic neuroinflammatory conditions mechanistically and pathologically linked to ischemic injury. Additionally, along with traditional writers such as P300, aminoacyl-tRNA synthetases AARS1 and AARS2 have been identified as enzymes that can regulate global lactylation levels. Their activity may be modulated by β-alanine administration, which alters lactate-mediated transcriptional programs and inflammatory responses [97]. These insights highlight the necessity of evaluating lactylation dynamics not only in neurons but also in glial subtypes, particularly microglia and astrocytes, as the cell-type-specific consequences of lactylation can differ significantly. Furthermore, this raises an important hypothesis: lactylation may be beneficial when transient, functioning as an adaptive response, but detrimental when persistent, potentially leading to chronic inflammation and secondary injury. The temporal window of lactylation activity during hypoxia and reperfusion may represent a critical therapeutic target.

### The therapeutic potential and possible targets of lactylation modifications in ischemic brain injury

Research on lactylation modifications in ischemic brain injury remains in its early stages, with only a limited number of foundational studies conducted. As a result, there have been no clinical trials examining drugs that specifically target lactylation modifications, nor have any drugs been developed with this focus. However, as the body of literature on lactylation modifications in ischemic brain injury expands, targeting these modifications presents significant potential for future therapeutic strategies in the treatment of brain injuries. Therefore, this study investigates potential targets for lactylation modifications in ischemic brain injury, establishing a foundation for future research in this area.

### Glycolytic enzyme inhibitors

One strategy involves targeting key enzymes in glycolysis to reduce lactate production and mitigate its accumulation in ischemic brain injury [98–100]. Experimental studies have demonstrated the potential of pharmacological agents that inhibit enzymes such as hexokinase, phosphofructokinase, and LDH. Specifically, inhibitors of HK2 have been shown to decrease lactate levels, which may aid in alleviating brain injury and enhancing neurological function [101–103]. Additionally, inhibiting pyruvate dehydrogenase kinase, which regulates the conversion of pyruvate to lactate, has proven effective in reducing lactate production and minimizing brain cell damage [104].

### MCT inhibitors

Inhibiting MCTs, particularly MCT1 and MCT4, has shown promise in reducing lactate efflux and mitigating brain injury in preclinical models of ischemic stroke. However, it is essential to recognize that complete inhibition of MCTs may lead to metabolic disturbances. The efflux of lactate through MCTs is vital for maintaining cellular pH balance and preventing lactate accumulation. While targeting MCTs may offer neuroprotective benefits in the context of ischemic brain injury, the potential side effects of total inhibition—such as metabolic acidosis and impaired cellular energy metabolism—must be carefully evaluated. Further research is required to determine the therapeutic window and identify the optimal degree of MCT inhibition that provides neuroprotection without causing significant metabolic dysregulation [105–107].

### Targeting lactylation or de-lactylation modifications

Targeting lactylation and de-lactylation modifications as a treatment for ischemic brain injury presents a promising strategy. However, before delving into the potential of these modifications, it is crucial to first investigate intracellular lactate levels. As a byproduct of glycolysis, lactate plays a vital role in regulating lactylation, making its intracellular concentration essential for the development of effective therapies. Recent research indicates that the role of lactylation modifications in ischemic brain injury is complex [86]. Lactylation can influence neuronal function by modulating both histone and non-histone proteins, potentially exacerbating pathological responses or enhancing the expression of neuroprotective genes. Consequently, lactylation modifications can exert dual effects, either contributing to brain injury or providing neuroprotection, depending on the underlying molecular interactions. Thus, further research is necessary to elucidate the mechanisms by which lactylation modifications affect ischemic brain injury and to assess their therapeutic potential.

## Discussion

Ischemic brain injury is a leading cause of severe disability and mortality worldwide. Although treatments such as thrombolytic therapy and mechanical thrombectomy have demonstrated effectiveness in the acute phase, challenges persist due to the limited treatment window and the brain injury that occurs following reperfusion. Consequently, there is an increasing emphasis on developing innovative therapeutic strategies for ischemic brain injury. In recent years, lactylation modifications have emerged as a novel epigenetic mechanism, garnering significant attention from researchers for their effects on cellular metabolism, immune regulation, and neuronal function. Lactylation involves the modification of proteins by lactate molecules through acylation, which alters their structure and function. As a byproduct of glycolysis, lactate plays a pivotal role in cellular energy metabolism and modulates protein function through lactylation, impacting cell survival, proliferation, and apoptosis.

Studies indicate that lactylation in ischemic brain injury is a complex mechanism that may either exacerbate damage by enhancing inflammation and cell death or provide protection by improving cellular metabolism and preventing neuronal death. Recent research suggests that the effects of lactylation modifications on ischemic brain injury are closely related to lactate levels, its origins, and the specific sites and molecules targeted by lactylation.

While some studies propose that exogenous lactate supplementation may alleviate ischemic brain injury, the impact of lactate varies significantly based on experimental conditions. In certain instances, lactate may confer protective benefits by regulating mitochondrial transfer and enhancing neuronal and astrocytic functions. However, excessive lactate accumulation and heightened lactylation modifications, particularly in specific cell types or target proteins, could exacerbate injury. Future research should focus on balancing lactate production with its modification effects while targeting the enzymes and molecules involved in lactylation modifications. Therapeutic strategies aimed at key enzymes in the glycolytic pathway, such as hexokinase and LDH, as well as lactate transporters like MCTs, show significant promise. These approaches may help mitigate lactate production and accumulation, thereby reducing the incidence of ischemic brain injury. However, therapies targeting lactylation modifications remain in early stages, with most research concentrated on animal models and cell experiments, lacking sufficient clinical evidence. Consequently, further investigation is warranted to explore the specific mechanisms of action of lactylation modification-based therapies across different forms of brain injury.

The dual role of lactylation modifications in ischemic brain injury offers valuable insights for ongoing research. While many aspects remain unclear, further exploration of the mechanisms underlying lactylation modifications and their interactions with other metabolic pathways could pave the way for more precise, targeted therapies for ischemic brain injury. Thus, the development of drugs aimed at lactylation modifications and associated metabolic pathways could provide new therapeutic options in clinical settings, leading to improved treatment outcomes and prognoses for patients suffering from ischemic brain injury.

**Table 1:** Summary of conflicting reports on lactate and lactylation modifications in ischemic brain injury

**Study**	**Model**	**Lactate/lactylation**	**Observed change**	**Mechanism/target**	**Effect on ischemic brain injury**
Yao et al. [84]	Adult rat CIRI model	Lactylation	↑ Global Kla (1003 sites)	Descriptive proteomics; unclear function	Undetermined
Sun et al. [85]	MCAO in mice	Lactylation	↑ MeCP2-K210/249 lactylation	↓ Pdcd4 and Pla2g6 → ↓ apoptosis	Protective
Zhou et al. [89]	MCAO in mice	Lactylation (ARF1)	↑ ARF1 lactylation (↓ LRP1)	Inhibits mitochondrial transfer → ↑ injury	Detrimental
LCP1 study [90]	MCAO in rats	Lactylation (LCP1)	↑ LCP1 lactylation	Promotes infarction, edema, apoptosis	Detrimental
Xiong et al. [91]	MCAO + LDHA KO	Lactylation	↓ Kla via LDHA/p300 inhibition	↓ neuronal death, ↓ glial activation	Protective (via inhibition)
Pan et al. [92]	MCAO in mice + EA	Lactylation	↓ Lactate and Kla levels	↓ astrocyte activation, ↓ neuronal death	Protective
Song et al. [93]	OGD-induced HUVECs + BHD	Lactylation	↓ H3K18la and pan-Kla	↓ Apaf-1, ↓ endothelial apoptosis	Protective
Sun [85] vs Xiong [91]	MCAO models	Lactate supplementation	[85] ↓ injury; [91] ↑ injury	Differ by intervention timing	Contradictory

Note: The table summarizes representative studies showing either protective or detrimental effects of lactate and lactylation in ischemic brain injury. Conflicting findings often arise from differences in intervention timing, target proteins, or cell-specific responses. Symbols: → direction of signal transmission; ↑ rising signal; ↓ falling signal. Abbreviations: Kla: Lysine lactylation; MCAO: Middle cerebral artery occlusion; CIRI: Cerebral ischemia-reperfusion injury; BHD: Buyang Huanwu Decoction; LDHA: Lactate dehydrogenase A; KO: Knockout; OGD: Oxygen-glucose deprivation.

However, contradictions in current literature exist, particularly regarding experimental design and the timing of interventions among studies. For instance, Sun et al. and Xiong et al. both explored the role of lactylation in ischemic brain injury, but their conclusions differ due to variations in sample size, timing of interventions, and experimental design. Sun et al.’s study did not implement randomization, had a small sample size, and lacked statistical control, potentially limiting the significance of their findings. Conversely, Xiong et al. employed a more complex intervention design, yet discrepancies in drug administration timing could have influenced outcomes due to inconsistent experimental conditions. Representative conflicting findings and study-level quality considerations are summarized in Table 1. These differences underscore the lack of standardization in current research and emphasize the importance of research quality in result interpretation.

Despite valuable insights into lactylation’s role in ischemic brain injury, contradictory results persist in the literature. These discrepancies likely arise from several underlying factors that warrant further exploration: (1) *Cell-type specificity:* Different cell types, such as neurons, astrocytes, and microglia, may respond differently to lactylation. For instance, lactylation may protect neurons while exacerbating inflammation in glial cells. The cellular context of lactylation significantly influences its functional outcomes, contributing to conflicting results. (2) *Timing of lactate surge:* The timing of lactate accumulation during ischemic brain injury may determine whether it has protective or detrimental effects. Early lactate accumulation may preserve cellular energy, while later-stage buildup could lead to acidosis and exacerbate neuronal injury. Thus, the timing of lactate surge is a key factor in reconciling conflicting data. (3) *Dose-dependent effects:* The concentration of lactate critically influences its impact on ischemic brain injury. Low lactate concentrations may promote cellular energy production and survival, while excessive accumulation can lead to toxicity, inflammation, and neuronal death. Future studies should define the threshold at which lactate becomes detrimental. (4) *Writer/eraser imbalance:* The balance between lactylation “writers” (enzymes that add lactate groups) and “erasers” (enzymes that remove lactate groups) is crucial in determining lactylation’s effects. Dysregulation of this balance may lead to contrasting outcomes; for instance, excessive lactylation or inadequate removal could promote neuroinflammation and worsen injury, while balanced lactylation may confer protection.

These factors underscore the complexity of lactylation’s role in ischemic brain injury and highlight the need for standardized experimental protocols that account for these variables. Understanding how these mechanisms interact will be key to resolving current discrepancies in the literature.

Future studies should prioritize standardizing experimental designs, employing appropriate sample sizes, and incorporating randomization and blinding. Further investigations should also explore the cell-type specificity of lactylation effects and the temporal dynamics of lactylation modifications to enhance understanding of lactylation’s dual role in ischemic brain injury.

## Conclusion

In conclusion, lactylation modification is a novel and critical epigenetic mechanism in the pathophysiology of ischemic stroke, exhibiting a context-dependent dual role. Its overall effect—whether neuroprotective or harmful—depends on a complex interplay of factors, including cell-type specificity, the timing and magnitude of the post-ischemic lactate surge, and the balance between lactylation writers and erasers. While therapeutic strategies that target glycolytic enzymes and lactate transporters (MCTs) show considerable promise by modulating lactate flux and subsequent lactylation, current evidence is predominantly preclinical. Future research should prioritize standardized experimental designs, clarify the precise spatiotemporal mechanisms of lactylation, and validate these findings in clinical settings to support the development of targeted, lactylation-based neuroprotective therapies for ischemic brain injury.
